# Genomic risk scores and oral contraceptive-associated ischemic stroke risk: a call for collaboration

**DOI:** 10.3389/fstro.2023.1143372

**Published:** 2023-09-14

**Authors:** Forrest Lin, Liisa Tomppo, Brady Gaynor, Kathleen Ryan, John W. Cole, Braxton D. Mitchell, Jukka Putaala, Steven J. Kittner

**Affiliations:** ^1^Department of Neurology, School of Medicine, University of Maryland, Baltimore, MD, United States; ^2^Department of Neurology, Helsinki University Hospital and University of Helsinki, Helsinki, Finland; ^3^Department of Medicine, School of Medicine, University of Maryland, Baltimore, MD, United States; ^4^Department of Neurology, Baltimore Veterans Affairs Medical Center, School of Medicine, University of Maryland, Baltimore, MD, United States; ^5^Geriatrics Research and Education Clinical Center, Baltimore Veterans Administration Medical Center, Baltimore, MD, United States

**Keywords:** oral contraceptives, stroke, genetics, risk, polygenic risk scores, young adults

## Abstract

**Background:**

Oral contraceptives (OCs) are generally safe but vascular risk factors increase OC-associated ischemic stroke risk. We performed a case-control study to evaluate whether a genomic risk score for ischemic stroke modifies OC-associated ischemic stroke risk.

**Methods:**

The Genetics of Early-Onset Stroke study includes 332 premenopausal women (136 arterial ischemic stroke cases and 196 controls) with data on estrogen-containing OC use within 30 days before the index event (for cases) or interview (for controls). Using a previously validated genetic risk score (metaGRS) for ischemic stroke based on 19 polygenic risk scores for stroke and stroke-associated risk factors, we stratified our combined case-control sample into tertiles of genomic risk. We evaluated the association between OC use and ischemic stroke within each tertile. We tested if the association between OC use and ischemic stroke depended on the genomic risk of stroke using logistic regression with an OC use × metaGRS interaction term. These analyses were performed with and without adjustment for smoking, hypertension, diabetes, coronary heart disease, and body mass index.

**Results:**

After adjustment for vascular risk factors, the odds ratio of OC use was 3.2 (1.7–6.3) overall and increased from the lower, middle, and upper tertile of genomic risk from 1.6 (0.5–5.4) to 2.5 (0.08–8.2) to 13.7 (3.8–67.3) respectively, and a *p*-value for interaction of 0.001.

**Conclusions:**

Our results suggest that genomic profile may modify the OC-associated ischemic stroke risk. Larger studies are warranted to determine whether a genomic risk score could be clinically useful in reducing OC-associated ischemic stroke.

## Introduction

Current recommendations advise against OC use in women with specific clinical conditions or risk factors, such as women 35 years of age or older who smoke 15 cigarettes or more daily (Medical Eligibility Criteria for Contraceptive Use, 2015). However, although the risk is lower, vascular disease also occurs in women taking estrogen-containing OCs without these contraindications. That genetic factors may contribute to the risk of OC-associated thrombotic events was first suggested in 1969 by the observation that venous thromboembolic disease events were more common in women with blood groups A, B, and AB compared to blood group (Jick et al., 1969). The genetic locus coding for blood groups has since been found to be a risk factor for thrombotic events, including stroke (Williams et al., 2013; Malik et al., 2018) and particularly early-onset ischemic stroke (Jaworek et al., 2022). It is also known that other genetic prothrombotic factors, such as factor V Leiden mutation and the prothrombin mutation G20210A increases the thrombotic risk of OC use (Vandenbroucke et al., 1994; Martinelli et al., 1999). With the advent of large genome-wide association studies of vascular disease and vascular risk factors, it has been possible to generate polygenic risk scores for risk of thrombosis, but there are few data on whether such scores appreciably modify the vascular risk of OCs, including the risk of ischemic stroke.

The aim of our study was to evaluate whether genetic predisposition for ischemic stroke, measured by a validated genomic risk score, modifies OC-associated ischemic stroke risk. This project was undertaken as a first step toward identifying women without contraindications to OC use who have a genetically-mediated OC-associated stroke risk similar to women with established contraindications, which would have important future implications for stroke prevention in young women.

## Methods

### Study cohort

The Genetics of Early Onset Stroke Study is a population-based case-control study designed to identify genetic determinants of early-onset arterial ischemic stroke and to characterize interactions with environmental risk factors. Stroke cases aged 15–49 years old at the time of ischemic stroke were recruited from the greater Baltimore-Washington area over 4 time periods between 1992 and 2008, along with age and sex matched controls (Hamedani et al., 2013). Study participants provided information from a standardized interview on age, height and weight, current smoking and current OC use, history of hypertension, diabetes, and coronary artery disease. Current smoking status and OC use were defined as use within 1 month before the event for cases and at a comparable reference time for controls (Hamedani et al., 2013). The study was approved by the University of Maryland at Baltimore Institutional Review Board, and all participants gave written informed consent.

The analyses in the present study were limited to 332 premenopausal women of European genetic ancestry with available data on estrogen-containing OC use. Cases and controls on progestin only OCs or on OCs of unknown type were excluded from analysis. Only participants of European ancestry were included because the genomic risk score was derived and validated in this population (Abraham et al., 2019).

### Genomic risk score and analysis

Study subjects were genotyped with the Illumina 1M array and imputed using the TOPMed reference panel on the University of Michigan Imputation Server, as previously described (Hamedani et al., 2013).

We used a previously validated genomic risk score (metaGRS) for ischemic stroke that is based on 19 polygenic risk scores for stroke and stroke-associated risk factors and validated in nearly 400,000 subjects from the UK BioBank (Hamedani et al., 2013). The metaGRS score was calculated based on the weighted sum of the allele dosages of 3,196,559 SNPs multiplied by their corresponding effect sizes.

We stratified our sample of women, inclusive of both cases and controls, into tertiles of genomic risk based on their metaGRS scores. The association between OC use and ischemic stroke was calculated within each tertile in two ways. First, we evaluated the unadjusted association of OC use with stroke risk and tested if the association between OC use and ischemic stroke depended on genomic risk of stroke using logistic regression with the 2 main effects of OC use and metaGRS, and an OC use × metaGRS interaction term. Secondly, we repeated the above analyses additionally adjusting for smoking, hypertension, diabetes, coronary heart disease, and body mass index.

## Results

Of the 59 OC users, 3 on progestin only OCs and 5 on OCs of unknown type were excluded from analysis. Of the 51 estrogen-containing OC users, the estrogen dose could not be ascertained with confidence in 13. The distribution of estrogen dose in the remaining 38 OC users was 20 mcg (2 users), 30 mcg (6 users), 35 mcg (20 users), 40 mcg (9 users), 50 mcg (1 user).

Characteristics of the 136 cases and 196 controls are shown in Table 1. Cases were more likely to report OC use than controls, more likely to report a history of current smoking, hypertension, diabetes, and coronary artery disease, and had a higher median body mass index.

**Table 1:** Risk factor characteristics by case control status.

	**Case**	**Control**	p-value^*^
Total N	136	196	
Median Age (IQR)	39 (32–44)	39 (35–43)	0.32
Median BMI (IQR)	25.9 (22.3–32.0)	24.2 (21.5–28.4)	0.02
Current smoker (%)	63/136 (46.3%)	42/196 (21.4%)	< 0.0001
Hypertension (%)	30/135 (22.2%)	14/196 (7.1%)	< 0.0001
Diabetes (%)	11/135 (8.1%)	3/195 (1.5%)	0.003
Coronary artery disease (%)	12/132 (9.1%)	5/196 (2.6%)	0.009
OC user (%)	28/136 (21.7%)	23/196 (11.7%)	0.03

IQR, interquartile range; BMI, body mass index; OC, oral contraceptive.

* p-values were calculated using chi-square for categorical variables and Wilcoxon rank sum test of medians for continuous variables.

The odds ratio for the association of the metaGRS with ischemic stroke was 4.4 (95% CI 0.6–37.0) unadjusted and 3.0 (95% CI 0.3–26.4) after adjustment for age and current smoking. Table 2 shows the odds ratio for oral contraceptive use stratified by tertile of metaGRS. In the unadjusted analysis, the point estimates for the odds ratios were 0.7, 1.6, and 11.5 in the lower, middle, and upper tertile of metaGRS and the test for interaction was statistically significant (*p* = 0.004). In the analysis adjusted for current smoking, hypertension, diabetes, coronary artery disease, and body mass index, the point estimates for the odds ratios were 1.6, 2.5, and 13.7 in the lower, middle, and upper tertile of metaGRS and the test for interaction was statistically significant (*p* = 0.001).

**Table 2:** Unadjusted and adjusted odds ratio for oral contraceptive use stratified by genomic risk score.

**Tertile based on metaGRS**	**OR for OC use (95% C.I.)**	**N OC users/N cases**	**N OC users/N controls**
**Unadjusted analysis**			
Upper tertile	11.5 (3.0–75.9)	14/47 (30%)	2/56 (4%)
Middle tertile	1.6 (0.5–4.5)	8/44 (18%)	9/73 (12%)
Lower tertile	0.7 (0.2–2.0)	6/45 (13%)	12/67 (18%)
All participants	2.0 (1.1–3.6)	28/136 (22%)	23/196 (12%)
***p*****-value for metaGRS** × **OC use interaction** = **0.004**			
**Adjusted analysis** ^*^			
Upper tertile	13.7 (3.8–67.3)	13/46 (28%)	3/63 (5%)
Middle tertile	2.5 (0.08–8.2)	8/41 (20%)	8/65 (12%)
Lower tertile	1.6 (0.5–5.4)	6/44 (14%)	12/67 (18%)
All participants	3.2 (1.7–6.3)	27/131 (21%)	23/195 (12%)
***p*****-value for metaGRS** × **OC use interaction** = **0.001**			

* adjusted for smoking, hypertension, diabetes, and coronary heart disease and body mass index.

OR, odds ratio; OC, oral contraceptive; C.I., confidence interval.

## Discussion

The main finding of our study is that the genomic risk score modified OC-associated stroke risk, even after adjustment for other vascular risk factors. In fact, the interaction was stronger in the risk-adjusted model because, paradoxically, the control group is enriched for women with vascular risk factors because women with vascular risk factors are less likely to be prescribed estrogen-containing OCs. Despite the strong evidence for a gene-environment interaction and the very high point estimate for the odds ratio in the upper tertile of genetic risk, the confidence interval for this risk estimate is very wide due to the small sample size of our study. Although estrogen dose was unknown for 25% of participants, 73% of OC users with known estrogen content were on low-estrogen pill with 35 mcg of estrogen or less.

There are currently a number of clinical situations where estrogen-containing OCs are considered to be contraindicated (Medical Eligibility Criteria for Contraceptive Use, 2015; Curtis, 2016). Prominent among these situations are women 35 of age and older who smoke more than 15 cigarettes per day. Although not stratified by age, the odds ratio for myocardial infarction among women who used estrogen containing OCs and smoked 25 or more cigarettes per day compared to nonsmokers and nonusers has been estimated at 32 (95% CI 12–81) (Rosenberg et al., 2001). There was no evidence that estrogen dose modified this marked risk. The WHO Collaborative Study of Cardiovascular Disease and Steroid Contraception found similar results for women smoking 10 or more cigarettes per day (Acute Myocardial Infarction Combined Oral Contraceptives, 1997). The point estimate for the excess stroke risk among OC users in the upper tertile of genetic risk is in the same range as these risks of myocardial infarction among smokers, although again with wide confidence intervals.

Our study has several strengths. The findings of a markedly excess risk only in the upper tertile of genetic risk is unlikely to be explained by differential reporting of OC use by cases and control. Thus, information bias cannot explain our findings. Similarly, case-control selection bias cannot explain our findings.

Our analysis has several limitations. First, the most critical limitation lies in the small sample size. Larger sample size will enable analyses with both more metaGRS strata and tighter confidence intervals. It is likely that only the highest 5% of genetic risk will be clinically significant and it will be important to estimate this risk with precision. Larger sample sizes will also allow sensitivity analyses based on the estrogen dose. Second, our study population is restricted to women of European ancestry from a small geographical region. Extending analyses into more diverse populations is important. This will be possible once a metaGRS based on other ancestries is available. Third, the current metaGRS does not include a polygenic risk score for deep venous thrombosis. We have previously shown that polygenic risk for deep venous thrombosis is more strongly associated with early-onset ischemic stroke compared to later onset ischemic stroke, emphasizing that thromboembolic mechanisms likely play a more central role in early-onset ischemic stroke compared to later onset ischemic stroke (Hamedani et al., 2013). Considering OC's impact on both venous and arterial thrombosis, adding a venous thrombosis polygenic risk into the model may further improve risk stratification. Finally, current polygenic risk scores only include common genetic variation; future genetic risk scores may include rare variants with larger effect sizes.

Taken together, the results of our exploratory analysis highlight the need for international collaboration to generate sufficient sample sizes to determine whether a genomic risk score could be clinically useful in reducing OC-associated ischemic stroke risk. When genetic sequence information becomes a routine part of the medical record, genetic risk stratification could influence future OC prescribing guidelines and reduce stroke in young women.

## Data availability statement

The demographic and genetic data utilized in this study are available via request from the database of Genotypes and Phenotypes (dbGaP) at https://www.ncbi.nlm.nih.gov/gap/ Search Study Term: phs000292.v1.p1. Additional data utilized in this study are available upon reasonable request to the corresponding author, Dr. Steven J. Kittner at email: skittner@som.umaryland.edu, subject to approval by the University of Maryland Institutional Review Board and Human Research Protections Office - email: hrpo@umaryland.edu; (620 W. Lexington St., Second Floor, Baltimore, MD 21201 - Phone: 410-706-5037).

## Ethics statement

The studies involving humans were approved by University of Maryland Institutional Review Board and Human Research Protections Office–email: hrpo@umaryland.edu; (620 W. Lexington St., Second Floor, Baltimore, MD 21201–Phone: 410-706-5037). The studies were conducted in accordance with the local legislation and institutional requirements. The participants provided their written informed consent to participate in this study.

## Author contributions

FL, KR, and BG conducted the analyses. LT wrote the first draft of the manuscript. SK and BM contributed to the conception and design of the study. All authors contributed to manuscript revision, read, and approved the submitted version.
